# Relation between leg extension power and 30-s sit-to-stand muscle power in older adults: validation and translation to functional performance

**DOI:** 10.1038/s41598-020-73395-4

**Published:** 2020-10-01

**Authors:** Julian Alcazar, Rikke S. Kamper, Per Aagaard, Bryan Haddock, Eva Prescott, Ignacio Ara, Charlotte Suetta

**Affiliations:** 1 GENUD Toledo Research Group, Universidad de Castilla-La Mancha, Toledo, Spain; 2 CIBER of Frailty and Healthy Aging (CIBERFES), Madrid, Spain; 3 Geriatric Research Unit, Department of Geriatric and Palliative Medicine, Bispebjerg-Frederiksberg University Hospital, Copenhagen, Denmark; 4 Institute of Sports Science and Clinical Biomechanics, University of Southern Denmark, Odense, Denmark; 5 Department Clinical Physiology, Nuclear Medicine & PET, Rigshospitalet-Glostrup University Hospital, Copenhagen, Denmark; 6 Department of Cardiology, Bispebjerg-Frederiksberg University Hospital, Copenhagen, Denmark; 7 Copenhagen City Heart Study, Frederiksberg University Hospital, Copenhagen, Denmark; 8 Geriatric Research Unit, Department of Medicine, Herlev-Gentofte University Hospital, Copenhagen, Denmark; 9 CopenAge - Copenhagen Center for Clinical Age Research, University of Copenhagen, Blegdamsvej 3, 2200 Copenhagen, Denmark

**Keywords:** Medical research, Geriatrics, Ageing

## Abstract

This study aimed to assess the validity and functional relevance of a standardized procedure to assess lower limb muscle power by means of the 30-s sit-to-stand (STS) test when compared to leg extension power (LEP), traditional STS performance and handgrip strength. A total of 628 community-dwelling older subjects (60–93 years) from the Copenhagen Sarcopenia Study were included. Physical performance was assessed by the 30-s STS and 10-m maximal gait speed tests. Handgrip strength and LEP were recorded by a hand-held dynamometer and the Nottingham power rig, respectively. STS muscle power was calculated using the subjects’ body mass and height, chair height and the number of repetitions completed in the 30-s STS test. We found a small albeit significant difference between LEP and unilateral STS power in older men (245.5 ± 88.8 vs. 223.4 ± 81.4 W; ES = 0.26; *p* < 0.05), but not in older women (135.9 ± 51.9 vs. 138.5 ± 49.6 W; ES = 0.05; *p* > 0.05). Notably, a large positive correlation was observed between both measures (r = 0.75; *p* < 0.001). Relative STS power was more strongly related with maximal gait speed than handgrip strength, repetition-based STS performance and relative LEP after adjusting for age (r = 0.53 *vs* 0.35–0.45; *p* < 0.05). In conclusion, STS power obtained from the 30-s STS test appeared to provide a valid measure of bilateral lower limb power and was more strongly related with physical performance than maximal handgrip strength, repetition-based STS performance and LEP.

## Introduction

In aged adults, skeletal muscle power has been demonstrated to be a stronger predictor of functional limitations compared to other physical capabilities such as muscle strength or maximal aerobic capacity^1,2^. In addition, maximal muscle power has been observed to decline from an earlier age and at a faster rate than muscle mass and strength^3^, and to be more strongly associated with mortality^4^.

Thus, the evaluation and routine monitoring of changes in muscle power with ageing and/or disuse should be strongly recommended in daily clinical practice with aged individuals. However, most of the testing protocols available in the literature require expensive equipment and/or can be highly time-consuming^5^, which often exclude the use of muscle power evaluation in large sample research studies (e.g. > 500 subjects) or in daily clinical practice.

Standing from a seated position is an essential activity of normal daily living and a vital prerequisite for bipedal walking^6^. The sit-to-stand (STS) test^7^ is an easy, rapid, and commonly used low-cost functional performance measure that evaluates the time taken to stand from a seated position a certain number of times or the number of repetitions undertaken in a given time period. STS performance is known to be associated with disability^8,9^, falls^10,11^, hip fracture^12,13^ and mortality^12,14^ among older adults. Although STS performance has traditionally been correlated with lower-limb muscle strength and power^15,16^, it does not represent per se an estimate of muscle strength or power, since the latter need to be expressed as N and W, respectively. Thus, time-based or repetition-based STS performance should remain as an independent and relevant measure of functional capacity, while more sophisticated procedures and advanced instruments are required to obtain yet other STS-related measures^17,18^. To enable a transition into direct power assessment, previous studies have evaluated STS muscle power by using force platforms^19–21^, linear position transducers^22–24^ or 3D accelerometers^25,26^. Still, as mentioned above, these procedures present significant economic and technical challenges^27^, which may restrain their applicability in large cohort studies or in a daily clinical setting.

In a recent study, STS muscle power was easily derived by collecting the subjects’ body mass and height as well as the chair height and the time needed to complete five STS repetitions^28^. This procedure was carefully validated against leg press muscle power values obtained with a linear position transducer. Nevertheless, it has been reported that 1 in 5 older adults cannot complete five successive STS repetitions^29^, which limits the feasibility of the 5-STS muscle power test in frail older subjects, whereas the 30-s STS protocol captures a wider range of older adults, allowing a score of zero in subjects who cannot complete a single STS repetition^16^.

Hence, the main goals of the present investigation were (i) to evaluate the validity of the newly proposed 30-s STS muscle power test against leg extension power (LEP) assessed using a previously validated multi-joint leg press setup; and (ii) to assess the association of STS muscle power to functional capacity when compared to other relevant muscle power and function measures.

## Results

### Validity of the 30-s STS muscle power test

Mean lower limb muscle power obtained by the STS test differed significantly from that measured by the Nottingham power rig for the entire cohort of participants (LEP ‒ unilateral STS power = 7.9 ± 60.0 W; *p* < 0.05; effect size (ES) ± 95% confidence intervals (CI) 0.10 ± 0.05) and for older men (LEP ‒ unilateral STS power = 21.0 ± 73.0 W; *p* < 0.05; ES ± 95% CI 0.26 ± 0.09) (Table 1). By contrast, no differences between test results were observed in older women (LEP ‒ unilateral STS power = ‒ 2.6 ± 44.4 W; *p* > 0.05; ES ± 95% CI 0.05 ± 0.08).

**Table 1:** Mean lower limb muscle power assessed by the Nottingham power rig and the 30-s sit-to-stand test.

	All (n = 628)	Women (n = 346)	Men (n = 282)
Bilateral STS power (W)	294.3 ± 130.2	230.8 ± 82.6	372.3 ± 135.7
Unilateral STS power (W)	176.6 ± 78.1	138.5 ± 49.6	223.4 ± 81.4
LEP (W)	184.9 ± 89.4*	135.9 ± 51.9	245.5 ± 88.8*

*LEP* unilateral leg extension power measured by the Nottingham power rig, *STS power* sit-to-stand power; for derivation of unilateral STS power, please see “Material and methods”.

Group means ± SD.

*Significant differences between unilateral STS power and LEP (*p* < 0.05).

**Figure 1:**
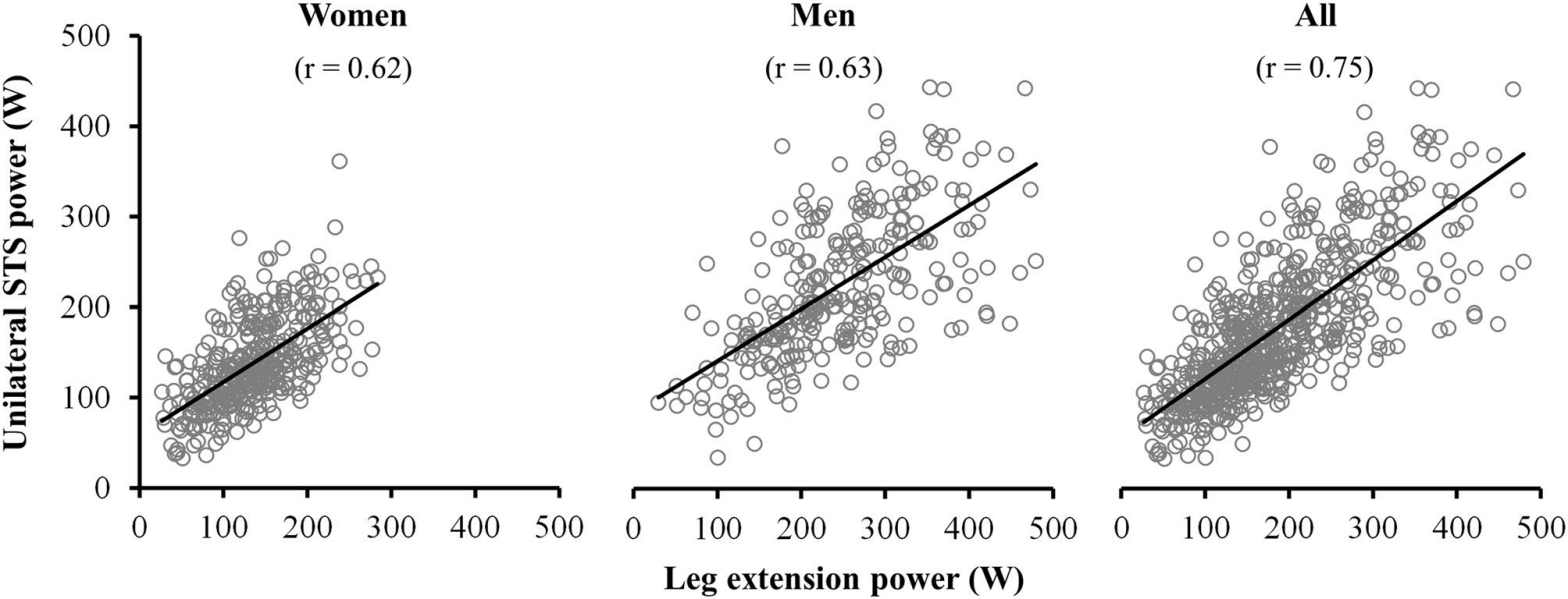
Pearson correlation plots for the association between unilateral lower limb muscle power measures obtained from the Nottingham power rig and the sit-to-stand test. *LEP* leg extension power, *STS* sit-to-stand.

**Figure 2:**
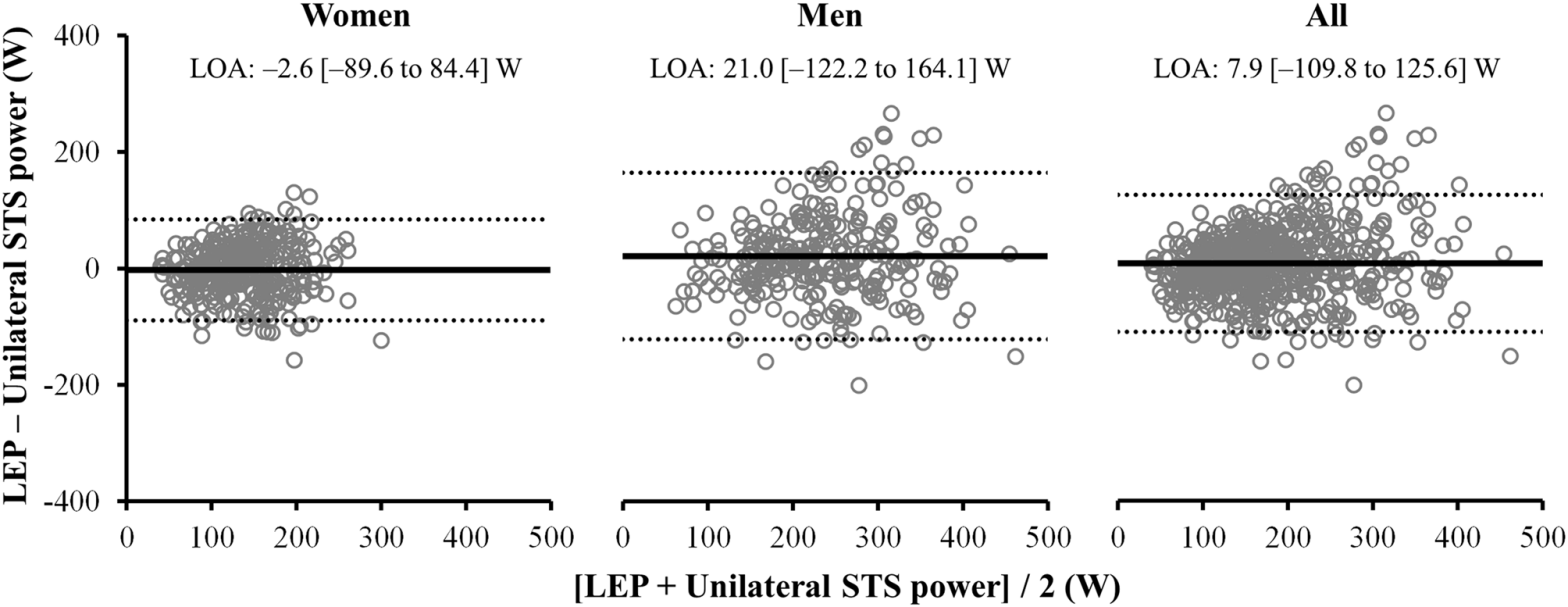
Bland–Altman plots for lower limb muscle power measures obtained from the Nottingham power rig versus the sit-to-stand test. *LOA* level of agreement, *STS* sit-to-stand.

The correlation between unilateral STS power and LEP values reached statistical significance when considering all participants (r = 0.75; standard error of the estimate (SEE) = 53.9; *p* < 0.001), as well as in women and men separately (r = 0.62, SEE = 41.0; and r = 0.63, SEE = 65.3; respectively, all *p* < 0.001) (Fig. 1). In addition, intra-class correlation coefficient (ICC) values were large in all the participants (ICC [95% CI] 0.85 [0.83–0.88]), and moderate-to-large when women and men were analyzed separately (ICC [95% CI] 0.76 [0.71–0.81] and 0.77 [0.71–0.82], respectively, all *p* < 0.001). Bland–Altman plots are displayed in Fig. 2. Despite the non-significant bias between the two power measures, a weak association between the difference and the average of both measures emerged when all participants were considered together (r = 0.15; *p* < 0.001), though not significant when women and men were evaluated separately (*p* > 0.05).

### Association of handgrip strength, 30-s STS performance, STS power and LEP with maximal gait speed

Correlations between maximal gait speed and handgrip strength, 30-s STS performance, relative STS power and relative LEP are displayed in Table 2. The unadjusted regression analyses showed that relative STS muscle power was more strongly correlated with maximal gait speed than handgrip strength, number of repetitions performed during the 30-s STS test and relative LEP in all the participants (r = 0.68 *vs* 0.53–0.61; *p* < 0.05). The correlation between maximal gait speed and relative STS power was also stronger than that observed with any of the other outcome variables when evaluated separately in women (r = 0.69 *vs* 0.50–0.63) and men (r = 0.63 *vs* 0.50–0.59), with differences reaching statistical significance for handgrip strength and relative LEP (both *p* < 0.05). Similar findings were observed after adjusting for age. The strength of the relationship between maximal gait speed and relative STS power was significantly higher when (i) compared with that of handgrip strength, 30-s STS performance and relative LEP in all the participants (r = 0.53 *vs* 0.35–0.45; *p* < 0.05), and (ii) compared with handgrip strength and relative LEP separately in men (r = 0.51 *vs* 0.26–0.37) and women (r = 0.49 *vs* 0.29–0.33) (all *p* < 0.05).

**Table 2:** Unadjusted and adjusted regression analyses to identify determinants of maximal horizontal gait speed in the present cohort of elderly-to-old adults (60–93 years, n = 628).

Variable	Maximal gait speed					
	Unadjusted			Adjusted by age		
	r	(95% CI)	SEE	r	(95% CI)	SEE
**All**						
HG strength	0.53	(0.47‒0.59)	0.46	0.35	(0.28‒0.42)	0.41
30-s STS test	0.61	(0.55‒0.68)	0.42	0.45	(0.39‒0.51)	0.38
STS power_REL_	0.68	(0.63‒0.74)	0.39	0.53	(0.47‒0.59)	0.36
LEP_REL_	0.55	(0.48‒0.61)	0.45	0.36	(0.29‒0.43)	0.41
**Women**						
HG strength	0.60	(0.52‒0.68)	0.41	0.37	(0.27‒0.47)	0.37
30-s STS test	0.63	(0.54‒0.71)	0.40	0.44	(0.36‒0.52)	0.35
STS power_REL_	0.69	(0.61‒0.76)	0.37	0.51	(0.43‒0.59)	0.34
LEP_REL_	0.50	(0.40‒0.59)	0.44	0.26	(0.17‒0.35)	0.39
**Men**						
HG strength	0.50	(0.40‒0.60)	0.47	0.29	(0.19‒0.41)	0.44
30-s STS test	0.59	(0.49‒0.68)	0.43	0.45	(0.35‒0.54)	0.39
STS power_REL_	0.63	(0.54‒0.72)	0.42	0.49	(0.40‒0.58)	0.39
LEP_REL_	0.51	(0.41‒0.61)	0.46	0.33	(0.22‒0.44)	0.43

*HG* handgrip, *STS* sit-to-stand, *LEP* leg extension power, *REL* relative to body mass, *SEE* standard error of the estimate.

## Discussion

The main study findings were: (1) no significant differences between lower limb muscle power obtained using the STS muscle power test and Nottingham power rig were found in older women, while small albeit significant differences existed in older men; (2) a strong linear relationship was observed between STS muscle power and corresponding LEP values; and (3) the association between maximal horizontal gait speed and relative STS muscle power was significantly stronger than that observed with either handgrip strength, 30-s STS performance or relative LEP.

### Validation of the 30-s STS muscle power test

Notably, small (ES = 0.26) but statistically significant differences between unilateral STS muscle power and LEP were observed in older men, but not in older women. These differences may have been caused by several factors. The Nottingham power rig evaluates mean unilateral leg muscle power during a brief concentric muscle action (< 1 s), while the power measure obtained from the STS test represents mean concentric bilateral leg muscle power exerted during the entire 30 s. Thus, in the present study unilateral STS muscle power was estimated based on bilateral deficit values previously reported for older adults^30^. In addition, an effective coordination of trunk, hip and knee extensors muscles is required during the STS task^31^, while the leg extension muscle action in the Nottingham power rig allows a more isolated work of the leg skeletal muscles. Differences in the results observed in men and women may arise from sex-related differences in anthropometric characteristics not reflected by the STS power test, which should be clarified in future experimental studies. In any case, a high correlation (r = 0.75) between STS power and LEP values was found, which was stronger than that observed between repetition-based STS performance and LEP values (r = 0.40). Notably, the present association between 30-s STS power and leg muscle power obtained with the Nottingham power rig was similar to that previously reported between 5-STS power and leg press muscle power (r = 0.72)^28^. Similar or lower correlation values between validated procedures to assess muscle power have previously been reported (force plate recording of vertical ground reaction force during single STS repetitions vs. Nottingham power rig-derived LEP^21^; linear position transducer recording during single STS repetitions vs. Nottingham power rig-derived LEP^32^; and computerized leg press vs. Nottingham power rig-derived LEP vs. Wingate test^33^; all r = 0.57 to 0.65). Collectively, these past and present findings confirm that the STS muscle power test used in the present investigation represents a valid measure of leg muscle power production in older people.

### Functional relevance of 30-s STS muscle power

Because of the progressive ageing of the population and the concomitant increase in older people experiencing mobility limitations, a key objective is to develop functional tests that are simple, non-expensive, non-time-consuming, require only basic equipment, and are valid, reliable and repeatable^34^. Traditional STS testing has been demonstrated to be feasible and reliable in a large variety of both healthy cohorts^35^ and patient populations^36,37^. In addition, STS muscle power testing has been found to provide more clinically relevant measures compared with time-based STS performance in terms of demonstrating stronger relationships with physical function, cognitive function and muscle mass among older subjects^28^. Notably, we observed that relative STS power is more strongly correlated with maximal gait speed than handgrip strength, repetition-based STS performance and relative LEP. The superior predictive strength of STS power measures on horizontal gait performance may be due to the STS task requiring a more integrated combination of muscle strength, coordination and postural control^31,38^ than muscle strength/power tests such as handgrip strength and unilateral LEP (Nottingham power rig). Frailty is considered an emerging public health priority that is associated with disability, poor quality of life and elevated mortality in older people, and is substantially influenced by impairments in muscle function^39^. Consequently, inclusion of the STS muscle power test within currently available frailty scales^40^ could provide a useful tool to identify early stages of frailty. To detect a real change (i.e. minimum clinically important difference) in the 30-s STS test, previous reports have indicated that a change of at least 2 repetitions is required^41^. In the present study, the latter would represent a change in STS power of 29.2 W (95% CI 20.5–38.0 W) or 0.43 W kg^−1^ (95% CI 0.31–0.56 W kg^−1^) in older women, and 43.8 W (95% CI 27.8–59.7 W) or 0.52 W kg^−1^ (95% CI 0.34–0.70 W kg^−1^) in older men, which is very similar to available data obtained using 5-STS muscle power testing in old adults (28.4–40.5 W)^28^.

In a clinical perspective, impaired skeletal muscle power is a major contributor to the development of functional limitations and the onset of disability at old age^1, 2^. Thus, muscle power assessment should be a more common procedure in older people, as well as interventions aiming to improve muscle power in older people with impaired levels^42,43^. The STS power test employed in the present study appears to provide a feasible and reliable procedure in older adults, and the data reported in the present study and elsewhere^28^ clearly demonstrate this methodology to be valid and clinically relevant among older people. The methodology is easily adaptable to different versions of the STS test (and different seat heights). However, it is important to note that different versions of the STS test may not be interchangeable. Therefore, while the shortest versions (i.e. 5-STS and 30-s STS tests) would reflect the anaerobic power of the older subjects, longer versions (i.e. 1-min STS and 3-min STS tests) would be more closely associated to the subjects’ aerobic power and cardio-respiratory exercise tolerance^44,45^. Finally, the utilization of the STS muscle power test in the clinical setting may be indicated in those patients presenting or being at risk for low physical performance, in order to confirm or discard low relative muscle power as a contributor to impaired functional ability. If observing a low relative muscle power in a mobility-limited patient should preferably lead to the prescription of progressive resistance training^42,43^.

Among the limitations of the current study, equation-derived STS muscle power was not compared with instrument-derived STS muscle power (e.g. force plate-derived), which should be accomplished in future studies. Notably however, the Nottingham power rig has been specifically evaluated and recommended to assess muscle power in older people^49^. In addition, the present associations between STS muscle power and physical performance were assessed using a cross-sectional study design. Future longitudinal studies should evaluate the prognostic value of STS muscle power testing in relation to the incidence of mobility limitations and frailty in old adults.

## Conclusions and implications

The 30-s sit-to-stand muscle power test provided muscle power values that were comparable to values obtained using a validated instrument (Nottingham power rig). In addition, relative STS muscle power was more strongly associated with maximal gait speed than handgrip strength, repetition-based STS performance and relative LEP. The STS muscle power test proved to be a reliable, easy, inexpensive, and fast way to assess lower limb muscle power in clinical or other health/science-related settings.

## Methods

### Participants

A total of 628 older subjects (346 women and 282 men) participated in this investigation (Table 3). The sample was composed of older people (≥ 60 years old) participating in the *Copenhagen Sarcopenia Study*^46^, a population-based cross-sectional study that included men and women aged 20–93 years living in the Copenhagen metropolitan area (Denmark). Exclusion criteria included acute medical illness, surgery within the last three months, ongoing medication known to affect body composition and/or reporting any history of compromised ambulation or prolonged immobilization. Several physical assessments and tests were performed by the participants in the following order: anthropometrics, handgrip strength, maximal horizontal gait speed, maximal leg extension power and STS performance. All subjects gave their written informed consent and the study was performed in accordance with the Helsinki Declaration and approved by the Ethical Committee of Copenhagen (H-3-2013-124).

**Table 3:** Physical and functional characteristics of study participants.

	Women (n = 346)	Men (n = 282)	All (n = 628)	
	Mean ± SD	Mean ± SD	Mean ± SD	Range
Age (y)	73.0 ± 8.0	71.6 ± 7.1	72.4 ± 7.7	60.0‒93.0
Height (m)	1.63 ± 0.06	1.77 ± 0.07	1.70 ± 0.10	1.44‒1.93
Weight (kg)	67.6 ± 11.9	84.0 ± 14.4	75.0 ± 15.4	38.0‒137.5
BMI (kg m^−2^)	25.4 ± 4.4	26.6 ± 4.0	26.0 ± 4.3	16.9‒42.9
Gait speed (m s^−1^)	1.9 ± 0.5	2.1 ± 0.5	2.0 ± 0.5	0.5‒4.2
HG strength (kg)	24.8 ± 6.0	42.4 ± 9.1	32.7 ± 11.6	7.3‒72.7
30-s STS test (reps)	15.9 ± 5.3	17.3 ± 5.9	16.5 ± 5.6	4.0‒38.0

*BMI* Body Mass Index, *HG* handgrip, *STS* sit-to-stand, *SD* standard deviation.

### Anthropometrics and functional capacity

A stadiometer and scale device (Seca 711, Seca, Germany) was used to record the height and body mass of the participants while wearing light clothing and no shoes. Body Mass Index (BMI) was obtained from the ratio between weight and height squared (kg m^−2^). Functional capacity was evaluated by means of maximal horizontal gait speed. Subjects were asked to walk at their maximal safe walking pace over a 10-m distance^47^. Subjects were given strong verbal encouragement during the test and were instructed to continue 2 m beyond the 10-m distance to avoid everyone from stopping or slowing down before reaching the 10-m distance. The time to complete the task was recorded to the nearest 0.1 s, and then converted into velocity (m s^−1^).

### Sit-to-stand testing

The 30-s STS test involves recording the number of STS repetitions performed in 30 s. The subjects were allowed to try 1–2 times with and adequate resting period (30–60 s) before the definitive STS test was performed. The subjects were in the sitting position with arms crossed over the chest on a standardized armless chair (0.45 m seat height). After the cue “ready, set, go!”, the subjects started to perform STS repetitions as rapidly as possible from the sitting position with their buttocks touching the chair to the full standing position. Participants were allowed to stop if they felt exhausted. A stopwatch was started simultaneously with the “go!” cue and it was stopped when the 30-s time limit was reached. The total number of completed sit-to-stand maneuvers during the 30-s period was recorded. Strong verbal encouragement was given throughout the test. As described in detail elsewhere^28^, STS mean velocity (m s^−1^) was calculated as the vertical distance (m) covered by the body center of mass divided by the mean time (s) spent to complete the concentric (upward) phase of one STS repetition (Eq. (1)). Vertical displacement of body center of mass was approximated from the difference between standing leg length (0.5 body height)^28^ and the height of the chair. The time spent to complete the concentric phase of one STS repetition was calculated as half the duration of the entire test (30 s) multiplied by the total number of repetitions completed during the test (i.e. assuming that duration of the concentric and eccentric phases is similar^48^). Mean acceleration over the concentric displacement phase was zero since initial and final velocities always were zero. Therefore STS mean force (N) was calculated as the body mass displaced during the test (total body mass minus shanks and feet mass) (0.9 body mass) (kg)^28^ multiplied by *g* (9.81 m s^−2^) (Eq. (2)). Subsequently, STS mean muscle power (W) was calculated as the product of STS mean velocity and STS mean force (Eq. (3)). Relative STS mean muscle power (W kg^−1^) was calculated as the STS mean muscle power normalized to total body mass (Eq. (4)).

1 $$STS\; mean \;velocity = \frac{{\left[ {Height \times 0.5 - Chair \;height} \right]}}{{30 {\text{s}} \times n\; of\; reps^{ - 1} \times 0.5}}$$

2 $$STS\; mean\; force = Body \;mass \times 0.9\;{\text{g}}$$

3 $$STS \;mean \;power = \frac{{Body\, mass \times 0.9\,{\text{g}} \times \left[ {Height \times 0.5 - Chair\; height} \right]}}{{30 s \times n \;of\; reps^{ - 1} \times 0.5}}$$

4 $$Relative \;STS\; mean \;power = \frac{{0.9\;{\text{g}}\;\left[ {Height \times 0.5 - Chair\; height} \right]}}{{30 s \times n \;of\; reps^{ - 1} \times 0.5}}$$

### Assessment of mechanical muscle function

#### Handgrip strength

Maximal handgrip strength was assessed using a Jamar dynamometer (Sammons Preston Rolyan, Chicago, USA). Participants were seated in the upright position with the arm along their side, elbow flexed at 90° and the forearm supported by a horizontal surface. Strong verbal encouragement was given during each trial. The best of three attempts with each hand (with 30–60 s of rest in between) was chosen for further analysis.

#### Leg extension power

LEP was evaluated by means of the Nottingham power rig (Medical Engineering Unit, University of Nottingham Medical School, Nottingham, UK)^49^. This device measures unilateral power production of the leg extensors. The participants were familiarized with the test procedure in two warm-up trials and then instructed to push the pedal forward as hard and fast as possible. Then, the subjects performed at least 5 repetitions with a 30-s resting period between successive attempts. The test was performed separately on each leg and measurements were repeated for each limb until maximal power output could not be increased further. The participants were seated in an upright position with their arms folded across the chest, knees flexed having one foot resting on the floor, and the other foot positioned on the dynamometer pedal connected to a flywheel. After the cue “ready, set, go!”, the subjects performed one single unilateral leg extension as rapidly as possible. The final angular velocity of the flywheel was used to calculate the mean LEP during the push^49^. Strong verbal encouragement and visual feedback were provided to all study participants to ensure a maximal volitional effort. The highest LEP value was selected for further analysis. In addition, relative LEP was calculated as LEP normalized to total body mass (W kg^−1^).

### Statistical analysis

Data are presented as mean ± standard deviation (SD). In order to examine the level of agreement between STS-derived and power rig-derived power measures, unilateral STS muscle power was calculated considering that a 20% of bilateral deficit in muscle power production has been previously reported in older adults^30^.

Differences between unilaterally transformed STS power values and those obtained from the Nottingham power rig were assessed using Student’s t-testing for dependent samples. In addition, ES ([mean STS power ‒ mean LEP] pooled SD^−1^) with 95% CI were calculated to compare the two procedures^50^. Thresholds for interpreting the ES were as follows^51^: < 0.2 trivial, 0.2–0.6 small, 0.6–1.2 moderate, and > 1.2 large.

To assess the association between procedures used to assess power, ICC_2,1_ was also calculated and assessed as^52^: < 0.40 slight; 0.41–0.60 fair; 0.61–0.80 moderate; and > 0.80 large. In addition, Bland–Altman analyses were performed in order to evaluate the level of agreement between unilateral STS muscle power and LEP values.

Finally, bivariate linear regression analyses were performed to compare the strength of the relationship of handgrip strength, 30-s STS performance (i.e. number of repetitions), relative bilateral STS power and relative LEP values versus maximal horizontal gait speed. Relative, instead of absolute, power values were used in this analysis due to their stronger association to physical function^53^. Regression r-values were assessed as^51^: < 0.1 trivial; 0.10–0.29 small; 0.30–0.49 moderate; 0.50–0.69 large; 0.70–0.89 very large; and 0.90–1.00 extremely large. In addition, differences in regression r-values were assessed by comparison of 95% confidence intervals. A further regression analysis was conducted adjusting by age to assess the independent effect of handgrip strength, 30-s STS physical performance, relative bilateral STS power and relative LEP on maximal gait speed. SEE values are reported for the linear regression analyses.

All statistical analyses were performed using SPSS v20 (SPSS Inc., Chicago, Illinois) with the level of significance set at α = 0.05 using two-tailed testing.

## Data Availability

The data that support the findings of this study are available from the corresponding author on reasonable request.
